# A distribution-aware semi-supervised pipeline for cost-effective neuron segmentation

**DOI:** 10.1016/j.isci.2025.114507

**Published:** 2025-12-19

**Authors:** Yanchao Zhang, Hao Zhai, Jinyue Guo, Jing Liu, Qiwei Xie, Hua Han

**Affiliations:** 1 State Key Laboratory of Brain Cognition and Brain-inspired Intelligence Technology, Institute of Automation, Chinese Academy of Sciences, Beijing 100190, China; 2 School of Future Technology, University of Chinese Academy of Sciences, Beijing 101408, China; 3 School of Artificial Intelligence, University of Chinese Academy of Sciences, Beijing 101408, China; 4 Research Base of Beijing Modern Manufacturing Development, Beijing University of Technology, Beijing 100124, China

**Keywords:** health sciences

## Abstract

Semi-supervised learning offers a cost-effective approach for neuron segmentation in electron microscopy (EM) volumes. This technique leverages unlabeled data to regularize supervised training for robust neuron boundary prediction. However, distribution mismatch between labeled and unlabeled data, caused by limited annotations and diverse neuronal structures, limits model generalization. In this study, we develop a distribution-aware pipeline to address the inherent mismatch issue and enhance semi-supervised neuron segmentation in EM volumes. At the data level, we select representative sub-volumes for annotation using an unsupervised measure of distributional similarity, ensuring broad coverage of neuronal structures. At the model level, we encourage consistent predictions across mixed views of labeled and unlabeled data. This design prompts the network to align feature distributions and learn shared semantics. Experiments on diverse EM datasets demonstrate the effectiveness of our method, which holds the potential to reduce proofreading demands and accelerate large-scale connectomic reconstruction efforts.

## Highlights

• Addressing the distribution mismatch inherent in semi-supervised neuron segmentation
• An unsupervised heuristic for selecting representative sub-volumes in EM data
• A novel semi-supervised framework using mixed-view consistency regularization
• Extensive experiments across diverse EM imaging modalities and voxel resolutions

## Graphical abstract

**Figure:**
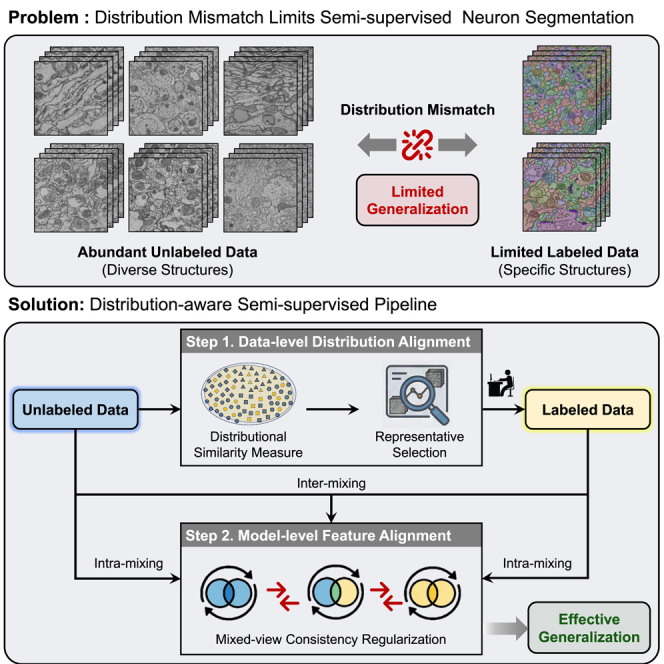

## Introduction

Connectomics aims to uncover brain mechanisms and intelligence by reconstructing and interpreting neural circuits at the synaptic level.^1^ Currently, only volume electron microscopy (EM), with its nanoscale spatial resolution, supports imaging the intricate morphology of neurons and individual synapses in brain tissue.^2^^,^^3^ However, this technique generates massive volumetric data ranging from several hundred terabytes to petabytes,^4^^,^^5^^,^^6^^,^^7^^,^^8^ which calls for automated methods to accelerate data processing and analysis. Therefore, efficient and accurate neuron segmentation from EM volumes has become critical to advance the digitalization of the complete atlas of the nervous system.

Recent advances in learning-based neuron segmentation have shown promising results across various EM modalities and voxel resolutions.^9^^,^^10^^,^^11^^,^^12^^,^^13^ Typically, these methods rely on convolutional neural networks to predict neuron boundary descriptors, followed by supervoxel extraction and agglomeration to obtain the final segmentation. Despite their success, a fundamental challenge remains: the high heterogeneity of neuronal structures in EM volumes requires substantial labeled data to train generalizable supervised models. However, obtaining such annotations is extremely costly and labor intensive. For example, the *Drosophila* hemi-brain segmentation model was trained on merely 0.003% of the entire dataset (0.8 GB out of 26 TB).^8^ The tension between data diversity and annotation scarcity poses a major barrier to effective model training.

Semi-supervised learning (SSL) has emerged as a promising paradigm to mitigate this challenge, which enhances model generalization by leveraging abundant unlabeled data. Related works in biomedical image segmentation have developed techniques, such as consistency regularization^14^^,^^15^^,^^16^ and self-training,^17^^,^^18^ to generate supervisory signals from unlabeled data. Notably, these methods typically assume that labeled and unlabeled data are drawn from the same distribution, thereby allowing unlabeled data to guide network optimization. In practical neuron segmentation, this assumption often fails due to the complexity of neuronal structures, large-scale unlabeled volumes, and limited annotation. Distribution mismatches can cause unreliable predictions on unlabeled data, resulting in suboptimal semi-supervised learning and degraded segmentation performance.

Addressing the inherent mismatch issue in semi-supervised neuron segmentation requires distribution-aware designs that explicitly model and align labeled and unlabeled data. At the data level, an intuitive yet underexplored direction is to select and annotate sub-volumes that effectively capture the distributional diversity of the entire dataset. Two main challenges, however, arise in this context. First, in a fully unsupervised setting, the absence of initial annotations makes it difficult to identify informative regions for labeling.^19^^,^^20^ Second, while diversity sampling^21^^,^^22^ is straightforward for discrete-instance datasets (e.g., ImageNet^23^), the spatial continuity of EM volumes makes it nontrivial to assess the representativeness of sub-regions with varying sizes. At the model level, a complementary direction is to align the feature spaces of labeled and unlabeled data within the semi-supervised framework. However, most existing approaches handle the two sets independently. As a result, distribution mismatch often leads to asymmetric feature learning, which limits generalization across neuronal structures.

In this work, we propose a distribution-aware pipeline that integrates complementary data-level and model-level designs to enhance neuron segmentation. Specifically, we first perform self-supervised learning on the unlabeled EM dataset to train an embedding network that generates semantically meaningful representations of local patches. Sub-volumes are represented by aggregating spatially adjacent patches in the embedding space, and a coverage-driven criterion is then applied to select representative ones for labeling. This process ensures that the selected sub-volumes faithfully capture the underlying data distribution, thereby mitigating distribution mismatch at the data level. Afterward, we train a segmentation model using selectively labeled sub-volumes and the unlabeled data in a semi-supervised manner. By adapting the spatial mixing strategy to volume EM data, we generate transitional inputs that bridge the feature distribution between datasets, facilitating shared semantic learning through mixed-view consistency regularization. This two-stage approach effectively reduces the distribution mismatch and improves semi-supervised learning, demonstrating practical value in cost-effective neuron segmentation. Our contributions are summarized as follows.

1. We propose a unified, distribution-aware semi-supervised pipeline that effectively mitigates mismatches between labeled and unlabeled distributions.
2. We develop an unsupervised, quantitative heuristic for selecting representative sub-volumes from EM data, capturing structural diversity under limited annotations.
3. We integrate spatial mixing into semi-supervised neuron segmentation to enforce mixed-view consistency and promote feature alignment.
4. We validate our approach on diverse EM datasets across species, modalities, and resolutions, demonstrating strong generalization and superior performance.

### Related work

#### Neuron segmentation

As an instance segmentation problem, neuron segmentation aims to assign a unique label to every voxel in the EM volumes that belongs to the same neuron. Advanced methods can be roughly categorized into two types: boundary-based and object-based methods. Specifically, boundary-based approaches first use 3D U-Net^24^ variants to predict descriptors for neuron boundaries, such as voxel affinity graph,^9^^,^^25^^,^^26^ dense voxel embeddings,^11^ and local shape descriptors.^13^ After over-segmentation via watershed transform, graph-based agglomeration is adopted to group supervoxels for instance results.^12^^,^^27^^,^^28^ In contrast, object-based approaches, as seen in some studies,^10^^,^^29^ extend the segmented area from the seed points to complete neurites iteratively.

While effective, large-scale neuron segmentation still demands years of manual proofreading before it can support reliable connectivity analysis.^6^ Enhancing the accuracy of automatic segmentation is therefore crucial for cost-effective connectomics. In this study, we adopt a boundary-based pipeline that employs a 3D CNN^9^ to predict a voxel affinity graph along the *z*, *x*, and *y* axes. To improve affinity predictions, we leverage both limited labeled data and abundant unlabeled data in a semi-supervised manner.

#### Semi-supervised segmentation

Labeled data typically constitute only a small fraction of the total imaging volume in practical reconstruction tasks. SSL offers a promising solution to this annotation bottleneck by leveraging unlabeled data to enhance model generalization. Self-training and consistency regularization represent two of the most prominent strategies in SSL for generating supervision signals from unlabeled data. Self-training assigns pseudo-labels to unlabeled samples, which are then combined with labeled data for iterative model retraining.^17^^,^^18^^,^^30^ Conversely, consistency regularization is based on the principle that predictions for unlabeled data should remain stable under various perturbations.^15^^,^^16^^,^^31^^,^^32^ Notably, SSNS-Net^14^ constitutes a pioneering effort in applying consistency regularization for neuron segmentation, demonstrating the promise of semi-supervised approaches in this domain.

SSL typically assumes that labeled and unlabeled data share the same underlying distribution.^33^^,^^34^ However, this often fails in neuron segmentation due to the intrinsic variability of neuron structures and limited annotation budgets. Despite its critical impact, distribution mismatch remains largely underexplored in this domain. This article proposes a distribution-aware semi-supervised pipeline that addresses the inherent mismatch through representative data selection and mixed-view consistency regularization.

#### Selective labeling

Selective labeling, a core focus of active learning, aims to annotate a valuable subset of an unlabeled dataset to optimize supervised learning under limited annotation budgets. Active learning methods are generally categorized as iterative active learning (ITAL) or one-shot active learning (OSAL), depending on whether samples are selected iteratively or all at once. ITAL generally involves iterative selection of informative samples based on uncertainty^35^^,^^36^^,^^37^ or diversity.^38^^,^^39^^,^^40^ The model is continually fine-tuned as new labeled data becomes available. However, ITAL methods seldom address the critical initial sample selection problem when no trained model exists. Moreover, their iterative cycle of training, inference, and annotation incurs high computational costs, especially for large-scale neuron segmentation. In contrast, OSAL selects all valuable samples in one shot before model training. Typically, these methods first estimate the unlabeled data distribution via unsupervised representation learning, projecting raw data into low-dimensional embeddings. They then employ customized strategies^19^^,^^20^^,^^21^^,^^41^^,^^42^ to identify representative and informative samples. Despite these advances, selective labeling for volumetric data such as EM volumes remains largely underexplored.

Given the spatial continuity and large size variability of neuronal structures, practical segmentation tasks typically rely on sub-volume annotations of varying sizes. However, existing OSAL methods typically operate on fixed-size, discrete instances, which restricts annotation flexibility. To address this limitation, we propose a tailored OSAL framework that selects informative sub-regions of flexible size directly from the continuous unlabeled EM volume, thereby improving distribution coverage under limited annotation budgets.

## Results

### A distribution-aware semi-supervised pipeline for neuron segmentation

**Figure 1:**
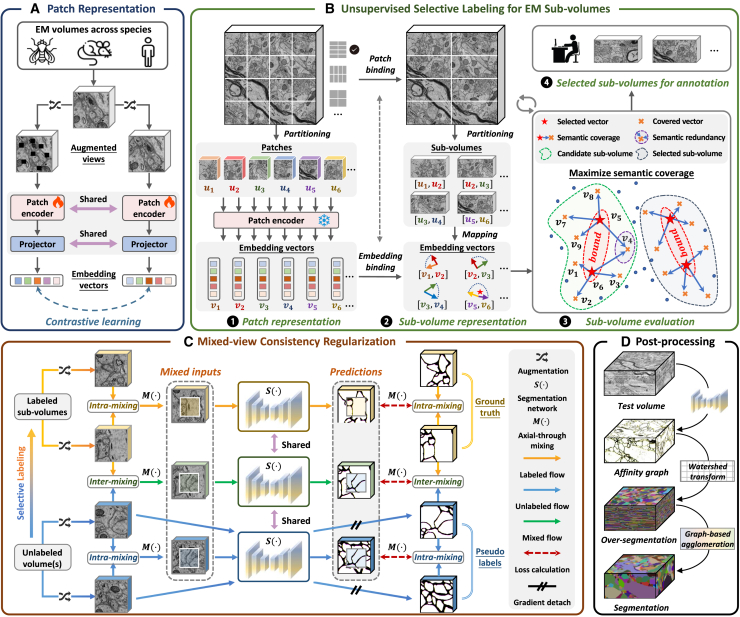
Overview of the distribution-aware semi-supervised pipeline for neuron segmentation (A) A 3D EM patch encoder is trained via contrastive learning across multiple data distributions. (B) After partitioning, the patch encoder maps unlabeled data to the embedding space. Then, a quantitative heuristic is designed to identify valuable sub-volumes as labeled data iteratively. This ensures broad distributional coverage across diverse neuronal structures. For illustration purposes, divisions are shown without overlap in this figure. (C) The selected sub-volumes and remaining unlabeled data are jointly exploited through intra- and inter-dataset mixing. Mixed-view consistency regularization is introduced to promote feature alignment and shared semantic learning across distributions. (D) The trained model first predicts a neuron affinity graph, followed by watershed over-segmentation and graph-based agglomeration for final segmentation.

We develop a semi-supervised pipeline that combines complementary data-level and model-level designs to mitigate distribution mismatch in neuron segmentation (Figure 1).

At the data level, we first perform self-supervised contrastive learning (SimSiam^43^) to train a patch encoder that maps EM patches into a low-dimensional embedding space, producing semantically meaningful representations (Figure 1A). Each sub-volume is viewed as an aggregation of spatially adjacent patches, with their embedding vectors jointly forming its compositional representation (Figure 1B). This formulation allows sub-volumes to vary in size and shape by defining different patch-binding schemes. On this basis, we implement a coverage-based greedy selection (CGS) strategy: each patch embedding is assumed to represent its *K*-nearest neighbors in Euclidean space, and candidate sub-volumes are iteratively selected based on maximal incremental coverage (Figure 1B). This method ensures that limited annotations capture the underlying distributional diversity of neuronal structures.

**Figure 2:**
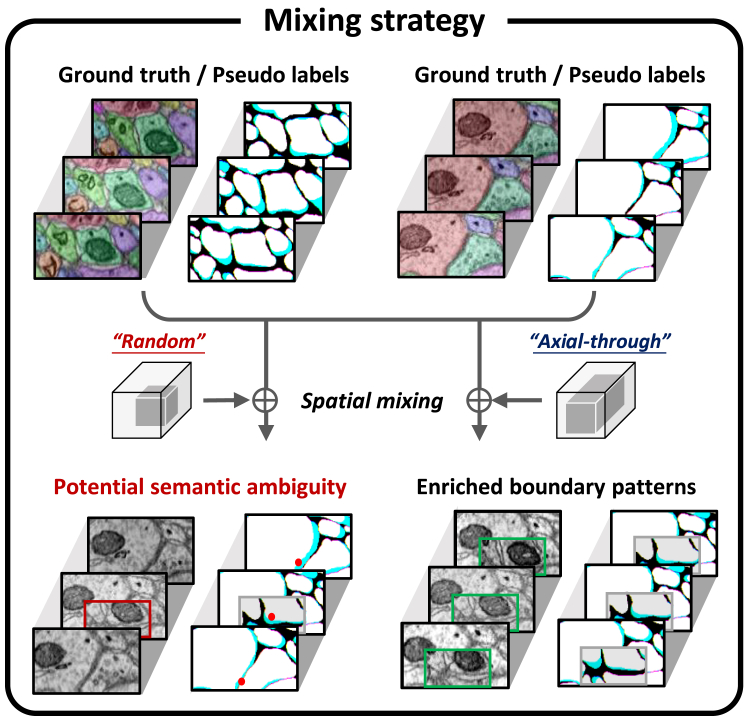
Axial-through spatial mixing for neuron segmentation The low axial resolution of anisotropic EM volumes can cause semantic ambiguity in the affinity graph when using standard random spatial mixing. We adopt an axial-through mixing strategy to preserve semantic continuity and prevent abrupt changes in the affinity graph.

At the model level, we introduce IIC-Net, a semi-supervised framework that leverages both labeled and unlabeled EM data through mixed-view consistency regularization. This framework incorporates both intra-dataset and inter-dataset mixing to promote feature alignment and shared semantic learning across distributions (Figure 1C). Specifically, IIC-Net employs an axial-through mixing strategy by adapting CutMix^44^ to 3D neuron segmentation, preserving semantic continuity and preventing ambiguity (Figure 2).

Together, these two components jointly form a unified distribution-aware pipeline to improve semi-supervised neuron segmentation. After training, the segmentation network predicts a voxel-wise neuron affinity graph for the test volume. The prediction is subsequently processed using watershed over-segmentation and graph-based agglomeration to generate neuron instances (Figure 1D). Detailed algorithmic procedures and implementation details are provided in STAR Methods.

### Effectiveness of coverage-based selective labeling for sub-volumes

#### Comparison with state-of-the-art methods

In this subsection, we evaluate the proposed selective labeling method (CGS)—the data-level component of our pipeline—on the AC3/AC4^45^ and CREMI^6^ datasets. This component constitutes the data-level design of our pipeline. All baseline methods are evaluated under the same annotation budget for each dataset to ensure a fair comparison. For patch-level selective labeling, we consider the following competitors: Random sampling, repeated four times with different random seeds, with the average reported as Random_P_; Equispaced sampling, denoted as Equispaced_P_; and one-shot active learning methods, including k-means,^46^ k median,^41^ farthest point sampling,^20^^,^^47^ representative annotation,^19^ and unsupervised selective labeling.^21^ For sub-volume selection methods, we compare CGS with random (Random_V_) and equispaced (Equispaced_V_) selection, as there is a lack of related research. Random selections are repeated four times, and the average results are reported. We also introduce two variants of CGS. The first, CGS_FD_, performs selection under a fixed-distance coverage function, where each patch covers neighbors within a fixed radius in the embedding space. The second, CGS_CS_, replaces the Euclidean distance with the negative cosine similarity to measure embedding similarity. Two commonly adopted agglomeration methods, Multicut^27^ and Waterz,^12^ are used for post-processing.

**Figure 3:**
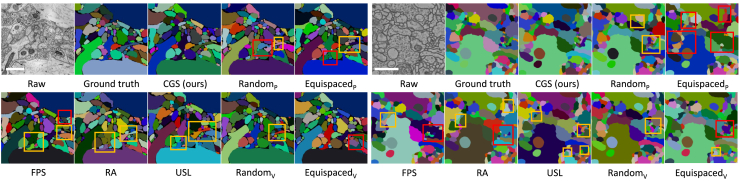
Visual comparison of selective labeling methods on the AC3/AC4 and CREMI datasets The red and yellow boxes represent merge and split errors, respectively. The scale bars represent 1 μm.

Quantitative results (Table 1) and qualitative visualizations (Figure 3) support the following conclusions. (1) CGS provides an efficient linear-time solution for sub-volume selection while reliably capturing the underlying data distribution. Across all tested datasets, CGS consistently achieves superior performance and demonstrates strong adaptability. (2) Sub-volume annotation substantially outperforms patch-level selection, highlighting its superiority for 3D neuron segmentation. Even randomly sampled sub-volumes surpass most patch-level baselines, which reveals the limitations of conventional OSAL methods on volumetric EM data. (3) The fixed-distance coverage variant, CGS_FD_, could ignore the variable density of patch embeddings, resulting in suboptimal coverage and reduced accuracy.

**Table 1:** The quantitative comparisons of selective labeling strategies on the AC3/AC4 and CREMI datasets

Methods			Multicut				Waterz			
			VI_*s*_↓	VI_*m*_↓	VI↓	ARAND↓	VI_*s*_↓	VI_*m*_↓	VI↓	ARAND↓
AC3/AC4	**Patch-level**	Random_P_	0.9042	0.3883	1.2925	0.1477	0.9822	0.3837	1.3659	0.1637
		Equispaced_P_	0.9459	0.3270	1.2729	0.1224	0.9441	0.3249	1.2690	0.1220
		k-means	0.9818	0.5333	1.5152	0.2147	0.9859	0.4220	1.4079	0.1401
		k-median	0.9826	0.3037	1.2863	0.1245	0.9467	0.3692	1.3159	0.1602
		FPS	0.9594	0.3583	1.3177	0.1225	0.9314	0.3769	1.3083	0.1177
		RA	0.9424	0.3091	1.2515	0.1158	0.9212	0.2999	1.2211	0.1193
		USL	0.8997	0.3507	1.2504	0.1201	0.8974	0.3678	1.2651	0.1288
	**Sub-volume**	Random_V_	0.8774	0.3256	1.2030	0.1182	0.8915	0.3417	1.2307	0.1432
		Equispaced_V_	0.8407	0.3430	1.1837	0.1189	0.8520	0.3986	1.2506	0.1499
		CGS_FD_	0.8733	0.2888	1.1622	0.1150	0.9000	0.2958	1.1958	0.1239
		CGS_CS_	0.8407	0.2800	1.1207	0.1156	0.8190	0.2993	1.1184	**0.1141**
		CGS	0.8546	0.2717	**1.1263**	**0.1103**	0.7705	0.3196	**1.0901**	0.1144
CREMI	**Patch-level**	Random_P_	1.0034	0.9599	1.9633	0.2280	1.2531	1.4793	2.7324	0.3742
		Equispaced_P_	1.0014	0.8144	1.8158	0.2026	1.0643	1.1739	2.2382	0.3161
		k-means	1.0656	0.8106	1.8761	0.2240	1.1551	1.2520	2.4070	0.3061
		k-median	0.9511	0.7897	1.7407	0.2034	1.1473	1.3617	2.5090	0.3919
		FPS	1.0391	0.8372	1.8763	0.1817	1.2892	1.3643	2.6706	0.3406
		RA	0.9810	0.7769	1.7579	0.1790	1.2552	1.5709	2.8261	0.4077
		USL	1.0014	0.8144	1.8158	0.2026	1.0643	1.1739	2.2381	0.3161
	**Sub-volume**	Random_V_	0.9775	0.7039	1.6814	0.1843	1.0961	1.2460	2.3421	0.3360
		Equispaced_V_	0.8838	0.7897	1.6735	0.1626	1.0758	1.0967	2.1725	0.3219
		CGS_FD_	0.9463	0.5881	1.5326	0.1503	1.0152	1.0728	2.0880	0.2595
		CGS_CS_	0.9400	0.5887	1.5287	0.1377	1.1105	0.9564	2.0670	0.2576
		CGS	0.9169	0.5675	**1.4843**	**0.1281**	0.9693	0.8520	**1.8208**	**0.1944**

Bold items indicate the highest-scoring results.

To validate the generalizability of CGS, we extend the selective labeling experiments to two additional self-supervised methods, SimCLR^48^ and SwAV.^49^ The results on the AC3/AC4 dataset with multicut post-processing (Table 2) consistently show that CGS achieves stable improvements, confirming its robustness across different embedding spaces. We further investigate the impact of sub-volume size on segmentation performance by comparing CGS with Random_V_. To ensure statistical reliability, averages are also computed over four random trials. The quantitative results (Table 3) reveal two key insights. First, CGS consistently outperforms random selection across all sub-volume sizes, demonstrating both effectiveness and robustness. Second, sub-volume size influences labeling efficiency under random selection. Smaller sizes may fail to capture morphological continuity, while larger ones limit the number of candidate regions, potentially reducing semantic coverage.

**Table 2:** Quantitative Comparisons on the AC3/AC4 dataset with different self-supervised methods

Methods	SimSiam		SimCLR		SwAV	
	VI↓	ARAND↓	VI↓	ARAND↓	VI↓	ARAND↓
**Patch-level Annotation**						

Random_P_	1.2925	0.1477	1.2925	0.1477	1.2925	0.1477
Equispaced_P_	1.2729	0.1224	1.2729	0.1224	1.2729	0.1224
k-means	1.5152	0.2147	1.3155	0.1272	1.3060	0.1238
k-median	1.3177	0.1225	1.2807	0.1239	1.3079	0.1269
FPS	1.2863	0.1245	1.3079	0.1251	1.2875	0.1215
RA	1.2515	0.1158	1.2346	0.1206	1.2669	0.1230
USL	1.2504	0.1201	1.2772	0.1211	1.2665	0.1219

**Sub-volume Annotation**						

Random_V_	1.2030	0.1182	1.2030	0.1182	1.2030	0.1182
Equispaced_V_	1.1837	0.1189	1.1837	0.1189	1.1837	0.1189
CGS	**1.1263**	**0.1103**	**1.1131**	**0.1047**	**1.1287**	**0.1101**

Random sampling and equispaced sampling are independent of the embedding space, so their performance remains consistent across self-supervised methods. Bold items indicate the highest-scoring results. The multicut algorithm is used for post-processing.

**Table 3:** Quantitative Comparisons on the AC3/AC4 dataset with different sub-volume sizes

Size	Methods	Multicut		Waterz	
		VI↓	ARAND↓	VI↓	ARAND↓
**Patch-level Annotation**					

18 × 160×160	Random_P_	1.2925	0.1477	1.3659	0.1637

**Sub-volume Annotation**					

18 × 220×220	Random_V_	1.2254	0.1432	1.2838	0.1538
	CGS	**1.1524**	**0.1138**	**1.1870**	**0.1170**
18 × 300×300	Random_V_	1.1958	0.1214	1.2127	0.1406
	CGS	**1.1484**	**0.1111**	**1.1241**	**0.1121**
18 × 380×380	Random_V_	1.2030	0.1182	1.2307	0.1432
	CGS	**1.1263**	**0.1103**	**1.0901**	**0.1144**
18 × 460×460	Random_V_	1.1620	0.1348	1.2445	0.1744
	CGS	**1.1024**	**0.1163**	**1.1210**	**0.1130**

Bold items indicate the highest-scoring results.

#### Visualization of CGS in the embedding space

**Figure 4:**
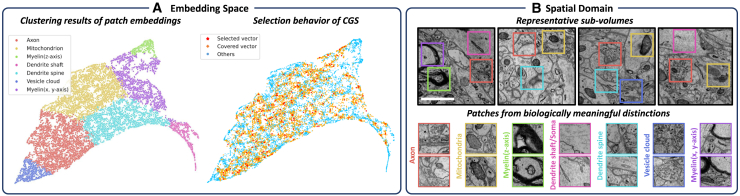
Visualization of CGS in the embedding space and the spatial domain (A) UMAP visualization of patch embeddings from the AC3/AC4 dataset. Spectral clustering reveals biologically meaningful groups, with dominant structures labeled in the legend. CGS-selected and covered patches are well distributed across the embedding space. (B) Spatial visualization of selected sub-volumes and example patches corresponding to distinct biological structures. The scale bars represent 1 μm.

We visualize the embedding space of EM patches from the AC3/AC4 dataset using UMAP (Figure 4A). Spectral clustering reveals biologically meaningful groups, including myelin, dendrite shafts or soma, dendritic spines, mitochondria, vesicle clouds, and axons. The patches selected and covered by CGS are broadly distributed across the embedding space, indicating that the strategy effectively captures the underlying structural diversity. Figure 4B further shows the CGS-selected sub-volumes and their example patches. This visualization demonstrates that the self-supervised embeddings effectively distinguish different neuronal structures and highlights the ability of CGS to select representative sub-regions from the raw EM data.

#### Impact of distribution mismatch on semi-supervised neuron segmentation

We evaluate the effect of distribution mismatch using IIC-Net on the AC3/AC4 and CREMI datasets. Building on the conclusions in Table 1, CGS-selected sub-volumes (denoted as *D*_*l*_) are well aligned with the unlabeled data (denoted as *D*_*u*_). To simulate distribution mismatch, we design two variants: (1) *D*_*u*_ is fixed, and *D*_*l*_ is replaced with randomly cropped sub-volumes ($\tilde{D_{l}}$). This process is repeated four times under the same annotation budget, and (2) *D*_*l*_ is fixed and the unlabeled set is randomly altered to form $\tilde{D_{u}}$. For AC3/AC4, we use unlabeled volumes from the Kasthuri dataset^45^; for CREMI, we sample unlabeled volumes from CREMI-B+/C+.^6^ Each setting also includes four randomly sampled subsets to ensure statistical reliability. The quantitative results presented in Table 4 underscore the detrimental impact of distribution mismatch on semi-supervised neuron segmentation. This subsection provides fundamental confirmation of the core motivation and necessity of our distribution-aware design.

**Table 4:** Impact of distribution mismatch in semi-supervised learning on the AC3/AC4 and CREMI datasets

Methods		Multicut		Waterz	
		VI↓	ARAND↓	VI↓	ARAND↓
AC3/AC4	CGS Selection (ours)				
	*D*_*l*_, *D*_*u*_	**0.9751**	**0.0922**	**1.0506**	**0.1002**
	Simulated Distribution Mismatch				
	$\tilde{D_{l}}$, *D*_*u*_	1.0694	0.1036	1.1207	0.1088
	*D*_*l*_, $\tilde{D_{u}}$	1.0629	0.1107	1.1189	0.1141
CREMI	CGS Selection (ours)				
	*D*_*l*_, *D*_*u*_	**1.3526**	**0.1122**	**1.5499**	**0.1416**
	Simulated Distribution Mismatch				
	$\tilde{D_{l}}$, *D*_*u*_	1.3767	0.1264	1.6631	0.2006
	*D*_*l*_, $\tilde{D_{u}}$	1.4313	0.1362	1.7227	0.1990

$\tilde{D_{u}}$ and $\tilde{D_{l}}$ denote modified unlabeled and labeled datasets, respectively, which are introduced to simulate distribution mismatch. Bold items indicate the highest-scoring results.

### Effectiveness of mixed-view consistency regularization for semi-supervised neuron segmentation

#### Comparison with state-of-the-art methods

In this subsection, we validate the superiority of the proposed semi-supervised framework (IIC-Net)—the model-level component of our pipeline—on the AC3/AC4 and CREMI datasets. Two supervised learning methods and eight semi-supervised learning methods are adopted for comparison. The supervised baselines include the following: (1) RSU-Net,^9^ using only labeled data in a supervised manner and (2) RSU-Net w/Mixing, incorporating axial-through spatial mixing for labeled data. The semi-supervised baselines include the following: (1) PseudoSeg,^50^ a classical semi-supervised approach that enforces consistency between unlabeled data and hard pseudo labels, (2) UA-MT,^16^ utilizing plausible predictions to guide consistency regularization, (3) SASSNet,^51^ employs an adversarial learning strategy to enforce a geometric shape constraint between the affinity graph of labeled and unlabeled data, (4) MC-Net,^32^ proposes mutual consistency regularization to encourage low-entropy predictions, (5) BCP,^52^ introduces bidirectional copy-paste method within a Mean Teacher framework, (6) MTANS,^53^ proposes a multi-scale Mean Teacher framework combined with adversarial training and shape-aware embedding, (7) DACL,^54^ guides contrastive learning with feature-space geometry and density, enhancing intra-class compactness, and (8) SSNS-Net,^14^ the first semi-supervised neuron segmentation framework with innovative network initialization and consistency regularization. Additionally, we consider two variants of the proposed method: IIC-Net without inter-dataset mixing (w/o inter-mixing) and IIC-Net without intra-dataset mixing (w/o intra-mixing).

**Figure 5:**
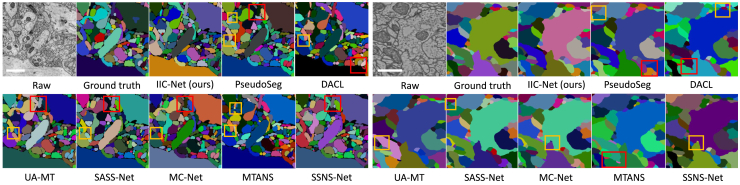
Qualitative comparison of semi-supervised methods on the AC3/AC4 and CREMI datasets The red and yellow boxes represent merge and split errors, respectively. The scale bars represent 1 μm.

For a fair comparison, we use consistent network architecture and augmentation strategies across all baseline methods. The quantitative results under two post-processing algorithms (Table 5) lead to three key observations. (1) Performing spatial mixing on labeled data can directly improve the segmentation performance in the supervised setting. This reveals the effectiveness of mixing strategies in promoting a better understanding of neuron boundaries. (2) The proposed IIC-Net achieves consistent improvements over all baselines and confirms the effectiveness of mixed-view consistency regularization. (3) Disabling intra-dataset mixing leads to a moderate performance decline, confirming its role in adapting the network to mixed inputs. A larger degradation occurs when inter-dataset mixing is removed, highlighting its pivotal function in mitigating the distribution mismatch and fostering shared semantic representations. A visual comparison of all methods is presented in Figure 5.

**Table 5:** The quantitative comparisons of semi-supervised methods on the AC3/AC4 and CREMI datasets

Methods			Multicut				Waterz			
			VI_*s*_↓	VI_*m*_↓	VI↓	ARAND↓	VI_*s*_↓	VI_*m*_↓	VI↓	ARAND↓
AC3/AC4	**Sup.**	RSU-Net	0.8546	0.2717	1.1263	0.1103	0.7705	0.3196	1.0901	0.1144
		RSU-Net w/Mix	0.7877	0.3482	1.1059	0.1061	0.7983	0.3144	1.1127	0.1031
	**Semi.**	PseudoSeg	0.8190	0.2687	1.0876	0.1073	0.8489	0.2934	1.1424	0.1270
		UA-MT	0.8018	0.2645	1.0662	0.0998	0.8588	0.2886	1.1473	0.1172
		MTANS	0.7805	0.3030	1.0835	0.1039	0.8194	0.2881	1.1075	0.1080
		SASSNet	0.7784	0.2914	1.0698	0.1143	0.8434	0.2467	1.0902	0.1086
		DACL	0.7832	0.2858	1.0691	0.1034	0.8207	0.2936	1.1143	0.1168
		MC-Net	0.7945	0.2592	1.0538	0.1082	0.8329	0.2390	1.0719	0.1049
		BCP	0.7864	0.2771	1.0634	0.1030	0.8202	0.3035	1.1238	0.1104
		SSNS-Net	0.7308	0.3166	1.0473	0.1025	0.7781	0.3113	1.0894	0.1165
		IIC-Net w/o Inter-mix	0.7395	0.2899	1.0293	0.1008	0.7732	0.2882	1.0614	**0.0960**
		IIC-Net w/o Intra-mix	0.7335	0.2792	1.0127	0.0937	0.8136	0.2559	1.0695	0.1072
		IIC-Net	0.6895	0.2855	**0.9751**	**0.0922**	0.7813	0.2693	**1.0506**	0.1002
CREMI	**Sup.**	RSU-Net	0.9169	0.5675	1.4843	0.1281	0.9693	0.8520	1.8208	0.1944
		RSU-Net w/Mix	0.8462	0.6004	1.4466	0.1336	0.9451	0.8743	1.8193	0.2034
	**Semi.**	PseudoSeg	0.9068	0.5406	1.4474	0.1362	0.9819	0.8659	1.8478	0.2289
		UA-MT	0.8966	0.5420	1.4386	0.1216	0.9718	0.7593	1.7311	0.1730
		MTANS	0.8859	0.5511	1.4370	0.1234	1.0132	0.7743	1.7874	0.1933
		SASSNet	0.8917	0.5552	1.4469	0.1265	0.9804	0.7711	1.7514	0.1815
		DACL	0.8888	0.5499	1.4387	0.1239	0.9761	0.7557	1.7317	0.1958
		MC-Net	0.8970	0.5409	1.4379	0.1235	0.9678	0.7537	1.7215	0.1942
		BCP	0.9134	0.5229	1.4363	0.1392	0.9279	0.7063	1.6342	0.1697
		SSNS-Net	0.8922	0.5329	1.4251	0.1217	0.9451	0.7229	1.6679	0.1633
		IIC-Net w/o Inter-mix	0.8472	0.5367	1.3838	0.1229	0.9323	0.7099	1.6400	0.1831
		IIC-Net w/o Intra-mix	0.8767	0.4986	1.3753	0.1258	0.8886	0.6707	1.5593	0.1576
		IIC-Net	0.8534	0.4992	**1.3526**	**0.1122**	0.8669	0.6830	**1.5499**	**0.1416**

Sup. and Semi. are abbreviations for supervised and semi-supervised learning. Bold items indicate the highest-scoring results.

#### Visualization of IIC-Net in the feature space

**Figure 6:**
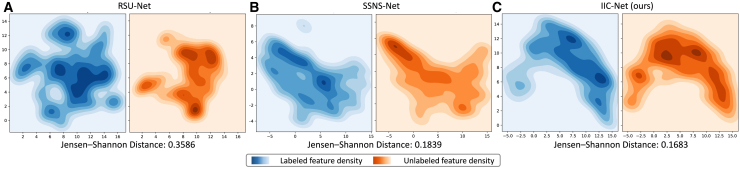
Kernel density estimations of deep features extracted from labeled and unlabeled data across different training methods

We visualize the feature distributions of the labeled and unlabeled datasets using kernel density estimation. To quantify their correspondence, we compute the Jensen-Shannon distance between the two distributions. As shown in Figure 6, semi-supervised models achieve closer alignment between labeled and unlabeled distributions than the supervised baseline. Notably, IIC-Net outperforms SSNS-Net and produces the most consistent feature representations. These results demonstrate that mixed-view consistency regularization effectively promotes feature alignment across datasets, supporting its role as the model-level design.

### Ablation study for the distribution-aware semi-supervised pipeline

To evaluate the effectiveness of the full distribution-aware pipeline, we conduct an ablation study across three datasets spanning different species, resolutions, and imaging modalities. These include AC3/AC4 (mouse; 29 × 6 × 6 nm ^3^), Hemi-brain (Drosophila; 8 × 8 × 8 nm ^3^),^8^ and AxonEM-H (human; 30 × 8 × 8 nm ^3^).^5^ The AC3/AC4 and AxonEM-H datasets are anisotropic volumes acquired by serial section EM. In contrast, the Hemi-brain dataset is isotropic and obtained using focused ion beam scanning EM. We progressively increase the annotation budget and assess performance under both supervised and semi-supervised settings. For selective labeling, we compare CGS with random sub-volume selection (Random ^∗^, best of four runs). For semi-supervised training, we use SSNS-Net as a representative baseline.

**Figure 7:**
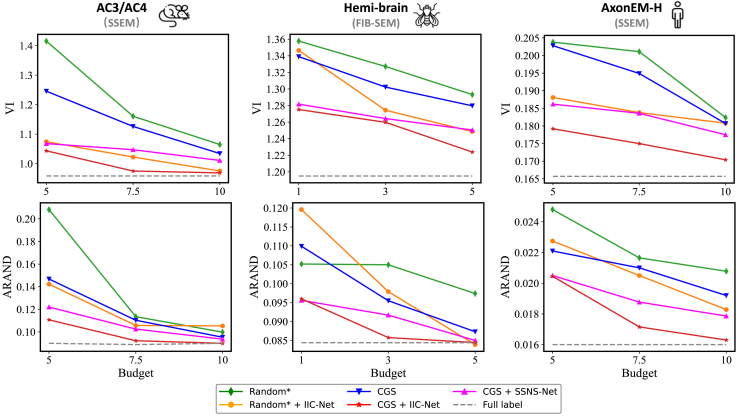
Quantitative Comparisons on the AC3/AC4, hemi-brain, and AxonEM-H datasets with different annotation budgets The AC3/AC4 and AxonEM-H datasets are anisotropic volumes acquired by SSEM, whereas the hemi-brain dataset is isotropic and obtained using FIB-SEM.

**Figure 8:**
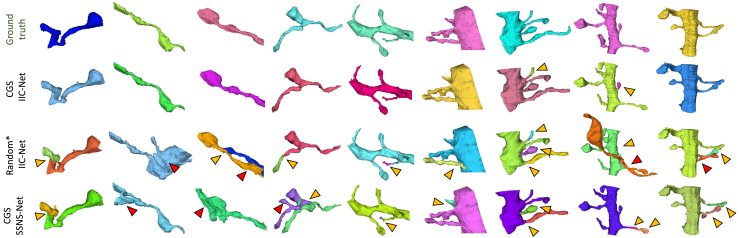
3D visual comparison of segmentation results on the AC3/AC4 dataset The red and yellow arrows represent merge and split errors, respectively.

Results with multicut post-processing are presented in Figure 7. Our pipeline, which integrates CGS-based sub-volume selection with IIC-Net training, consistently outperforms all baselines across datasets and annotation budgets. These consistent trends across species, resolutions, and imaging modalities suggest strong robustness and generalizability of our approach. Notably, on AC3/AC4, the pipeline achieves performance comparable to full supervision while using only 10% of the labeled data. The 3D visualizations in Figure 8 further illustrate the method’s ability to preserve structural continuity and produce topologically consistent segmentations. This advantage is especially evident in challenging regions rich in dendritic spines.

### Evaluation on large-scale EM data

In this subsection, we evaluate the proposed pipeline on the Kasthuri dataset^45^ to validate its practical utility in large-scale neuron segmentation. Quantitative results are summarized in Table 6. Under identical annotation budgets, IIC-Net consistently achieves higher segmentation accuracy compared with the supervised baseline. Notably, when using only 20% of the labeled data, our pipeline attains performance comparable to the fully supervised counterpart, demonstrating its strong data efficiency in large-scale settings. Considering the significant time and cost of expert proofreading, this finding highlights the potential of our framework to reduce annotation dependency and accelerate connectomic reconstruction.

**Table 6:** Evaluation on Kasthuri using the proposed semi-supervised pipeline

Budget	SL	SSL	VI↓	ARAND↓
10%	CGS	–	0.8540	0.1810
	CGS	IIC-Net	**0.8100**	**0.1741**
20%	CGS	–	0.8236	0.1795
	CGS	IIC-Net	**0.7842**	**0.1710**
100%	–	–	0.7751	0.1731
	–	IIC-Net	**0.7362**	**0.1632**

SL and SSL denote selective labeling and semi-supervised learning, respectively. Bold items indicate the highest-scoring results.

## Discussion

This work presents a distribution-aware semi-supervised pipeline that mitigates the distribution mismatch between labeled and unlabeled data in neuron segmentation. Specifically, coverage-based selective labeling ensures broad structural representation at the data level, while mixed-view consistency regularization promotes alignment of feature distributions at the model level. Together, these complementary components enable effective use of abundant unlabeled data and improve model generalization. Extensive experiments confirm the effectiveness of the proposed method and demonstrate significant improvements in segmentation accuracy. Beyond neuron segmentation, this distribution-aware design also provides a generalizable solution for other volumetric biomedical imaging tasks, offering a practical approach to handle limited supervision under data heterogeneity.

From a broader neuroscience perspective, converting terabyte-to petabyte-scale EM datasets into accurate connectomes remains heavily constrained by the extensive manual proofreading required.^5^^,^^6^ High-quality automated neuron segmentation is therefore essential for scalable and cost-effective connectomics.^13^^,^^55^ By improving segmentation accuracy under highly constrained annotation budgets, the proposed distribution-aware pipeline can meaningfully reduce proofreading demands and thereby help accelerate large-scale connectomic reconstruction efforts. In addition, more reliable and topologically consistent segmentation provides a critical foundation for downstream analyses, including cell-type classification, morphometric quantification, synaptic partner identification, and circuit modeling. Together, these improvements can facilitate a more systematic investigation of neural organization and information processing.

### Limitations of the study

Despite these advances, several limitations remain and indicate directions for further improvement. First, while coverage-based sub-volume selection provides broad structural representation, more adaptive strategies could be explored. For instance, dynamically adjusting sampling to local data density or incorporating biological prior knowledge may further improve labeling efficiency and reduce redundancy. Second, although semi-supervised learning benefits from large-scale pseudo-labels, the presence of noisy or ambiguous regions can still hinder performance. Developing more robust denoising techniques or confidence-aware weighting mechanisms for the neuron affinity graph could improve pseudo-label reliability and thereby enhance downstream training. Third, while one-shot active learning offers computational efficiency by minimizing labeling rounds, integrating it with iterative active learning may provide a more balanced trade-off. Such a hybrid strategy could leverage both distributional coverage and feedback from model performance. It would enable more targeted selection of informative sub-volumes and further improve segmentation accuracy in large-scale connectomic reconstructions.

## Resource availability

### Lead contact

Requests for further information and resources should be directed to and will be fulfilled by the lead contact, Hua Han (hua.han@ia.ac.cn).

### Materials availability

This study did not generate new materials.

### Data and code availability

• This article analyzes existing, publicly available data. The corresponding dataset sources and download links are listed in the key resources table.
• Codes and demos are available https://github.com/yanchaoz/SL-SSNS.
• Any additional information required to reanalyze the data reported in this article is available from the lead contact upon request.

## Acknowledgments

This work was supported by the Beijing Natural Science Foundation under grant 5254042, the 10.13039/501100001809National Natural Science Foundation of China under grant 32171461, and the STI 2030—major projects under grants 2021ZD0204500 and 2021ZD0204503.

## Author contributions

Conceptualization, Y.Z. and H.H.; methodology, Y.Z., H.Z., and J.G.; investigation, Y.Z., H.Z., and J.G.; writing – original draft, Y.Z.; writing – review & editing, Y.Z., J.L., and H.H.; supervision, J.L. and H.H.; funding acquisition, J.L. and H.H.; resources, Q.X.

## Declaration of interests

The authors declare no competing interests.

## STAR★Methods

### Key resources table

**Table:** 

REAGENT or RESOURCE	SOURCE	IDENTIFIER
**Software and algorithms**		

Neuroglancer	Github	https://github.com/google/neuroglancer
Multicut	Github	https://github.com/constantinpape/elf
Waterz	Github	https://github.com/funkey/waterz
SSNS-Net	Github	https://github.com/weih527/SSNS-Net
CGS algorithm	This paper	https://github.com/yanchaoz/SL-SSNS
IIC-Net	This paper	https://github.com/yanchaoz/SL-SSNS

**Other**		

AC3/AC4 dataset	Kasthuri et al.^45^	https://lichtman.rc.fas.harvard.edu/vast/AC3AC4Package.zip
SNEMI dataset	SNEMI3D/Grand Challenge	https://snemi3d.grand-challenge.org
CREMI dataset	CREMI organizers	https://cremi.org
AxonEM-H dataset	AxonEM/Grand Challenge	https://axonem.grand-challenge.org
Hemi-brain dataset	Sheridan et al.^13^	https://github.com/funkelab/lsd#notebooks
Kasthuri dataset	Kasthuri et al.^45^	https://neurodata.io/data/kasthuri15

### Experimental model and study participant details

Omitted as our study does not involve biological models.

### Method details

We develop a distribution-aware pipeline for cost-effective neuron segmentation that addresses the distribution mismatch between labeled and unlabeled EM data. Specifically, unsupervised selective labeling improves data-level distribution coverage, while mixed-view consistency regularization enhances feature alignment during semi-supervised training. The following subsections detail the components that constitute this innovative pipeline.

#### Unsupervised selective labeling for EM sub-volumes

Let *D*_*u*_ and *D*_*l*_ denote the unlabeled and labeled datasets, respectively, where *D*_*l*_ is selected under a given annotation budget. The objective is to identify and annotate representative sub-volumes that adequately capture the underlying distribution of *D*_*u*_. Our approach consists of two components: patch-level representation learning and coverage-based sub-volume selection.

##### Unsupervised representation learning for EM patches

We first employ self-supervised learning to train an embedding network that produces low-dimensional, semantically rich representations of 3D EM patches. To promote robust generalization across diverse neuronal patterns, the model is trained on EM datasets spanning three species: fly, mouse, and human. Specifically, our unsupervised training follows the SimSiam framework.^43^ It builds upon a Siamese architecture optimized to maximize the similarity between two augmented views of the same input in the embedding space (Figure 1A). We carefully apply perturbations that preserve semantic content, including reflections, rotations, Gaussian blurring, Gaussian noise, simulated misalignment, photometric changes, and random pixel masking. During training, each input volume $u\in R^{D\times H\times W}$ is sampled from the unlabeled dataset *D*_*u*_ and randomly augmented to produce two views, ${\tilde{u}}_{1}$ and ${\tilde{u}}_{2}$. These views are then passed through the Siamese networks consisting of two weight-sharing patch encoders *f*(·), two weight-sharing projectors *g*(·) and a predictor *h*(·). The parameters are updated by minimizing the symmetrized loss function:

(Equation 1) $$L_{Sim}=\frac{1}{2}N\left(h\left(g\left(f\left({\tilde{u}}_{1}\right)\right)\right),\underline{g\left(f\left({\tilde{u}}_{2}\right)\right)}\right)+\frac{1}{2}N\left(h\left(g\left(f\left({\tilde{u}}_{2}\right)\right)\right),\underline{g\left(f\left({\tilde{u}}_{1}\right)\right)}\right)\text{,}$$

where N(·,·) represents the negative cosine similarity between two representations. The underlined terms are treated as constants during backpropagation and do not contribute to gradients. The trained patch encoder *f*_*θ*_(·) subsequently serves as an embedding network that projects EM patches into an *L*-dimensional embedding space.

##### Coverage-based sub-volume selection

We propose a coverage-based heuristic to identify representative and informative sub-volumes within the embedding space. For clarity, we distinguish between a patch and a sub-volume: a patch is the smallest unit used for representation learning, whereas a sub-volume is an arbitrarily sized sub-region cropped from the EM volume. The sub-volume annotation strategy is more flexible and suitable for neuron segmentation. It addresses the limitations of patch-level annotation, which often fails to capture the spatial continuity and size variability of neuronal structures. Each candidate sub-volume is viewed as a structured aggregation of spatially neighboring patches, and their corresponding embeddings jointly define the representation of the sub-volume. This formulation allows sub-volumes to vary in size and shape by defining different patch-binding schemes. Carefully selected sub-volumes that cover diverse and typical patches provide a practical approximation of the underlying data distribution.

Formally, the unlabeled volume(s) can be represented as the universal set $U={\left\{v_{n}=f_{\theta }\left(u_{n}\right)\right\}}_{n=1}^{N}$. Here, each $u_{n}\in R^{L}$ is a 3D input patch extracted via sliding window partitioning, and *v*_*n*_ denotes its embedding vector. This process can be readily adapted to datasets of varying scales by adjusting the stride used in patch partitioning. As shown in Figure 1B, we represent candidate sub-volume(s) as a subset of *U*, denoted as the selected set $S={\left\{v_{m}|v_{m}\in U\right\}}_{m=1}^{M}$. It contains embeddings of spatially contiguous patches extracted from *U*.

Intuitively, the distance between the embedding vectors reflects the semantic similarity between their corresponding patches. We thus hypothesize that each selected vector in *S* could represent its *K*-nearest neighbors in *U*, referred to as its covered vectors. This relationship defines a notion of semantic coverage within the embedding space. Effective sub-volume selection should maximize semantic coverage to represent the data distribution and capture diverse neuronal morphologies (Figure 1B). However, semantic redundancy may arise when multiple vectors in *S* cover significantly overlapping embeddings in *U*. Such redundancy constrains the overall representational capacity. With this in mind, we introduce the constrained coverage rate (CCR) as a criterion to evaluate the semantic representativeness of the selected set *S*. Formally,

(Equation 2) $$V\left(v,U\right)=\left\{v_{n}\in U\;|\;{\left\|v_{n}-v\right\|}_{2}\leq {\left\|u-v\right\|}_{2},u\in U,\left|V\right|=K\right\}\text{,}$$

(Equation 3) $$C\left(S,U\right)=\underset{v_{m}\in S}{\cup }V\left(v_{m},U\right),\text{CCR}\left(S,U\right)=\frac{|C\left(S,U\right)|}{|U|}\text{,}$$

where ${\left\|\cdot \right\|}_{2}$ represent *l*_2_-norm, *V*(*v*,*U*) returns the *K*-nearest neighbors of *v* in *U*, and thus covered set *C* contains all the vectors in *U* that are within the *K*-nearest neighbors of at least one vector in *S*. CCR defines the coverage rate as the percentage of covered vectors in *U*. A higher CCR indicates that the selected sub-volumes cover a larger portion of the neuronal structures in the entire volume and thus better represent the overall data distribution.

Given an annotation budget, our objective is to select a subset *S*⊆*U* that maximizes the coverage rate CCR(*S*,*U*), subject to spatial constraints among the vectors in *S*. The optimal subset could theoretically be obtained by exhaustively evaluating all possible sub-volume combinations. However, this brute-force approach becomes computationally intractable as the dataset size and annotation budget grow. To address this challenge, we propose a coverage-based greedy selection (CGS) algorithm with linear time complexity, offering a practical and scalable solution for large-scale EM volumes. At each iteration, CGS scans the entire volume to generate candidate sub-volumes and extracts their corresponding embedding vectors from *U*. These vectors are provisionally added to the current set *S* to assess its incremental gain in semantic coverage. The sub-volume yielding the largest improvement is then selected for annotation. This process is repeated until the annotation budget is exhausted. Further details are provided in Algorithm 1.

Algorithm 1 **Coverage-based Greedy Selection for Sub-volumes (CGS)**

**Input:** unlabeled volume *D*_*u*_, patch encoder *f*_*θ*_(·), sub-volume sizes ${\left\{s_{i}\right\}}_{i=1}^{t}$

**Output:** selected sub-volumes *D*_*l*_

1 selected set: *S*←∅, universal set: *U*← patch-level embeddings extracted from *D*_*u*_ using *f*_*θ*_(·)
2 **for** *each sub-volume of size s*_*i*_∈*s*_1_,*s*_2_,…,*s*_*t*_ **do**
3 *best*_*subset*←∅,*best*_*rate*←-*∞*
4 **for** *each positionpin raw volume using sliding window of size s*_*i*_ **do**
5 *subset*← extract vectors from *U* with {*p*,*s*_*i*_}
6 coverage_rate←CCR(subset∪S,U)
7 **if** *coverage_rate* > *best_rate* **then**
8 *best*_*subset*←*subset*, *best*_*rate*←*coverage*_*rate*
9 **end**
10 **end**
11 S←S∪best_subset
12 **end**
13 *D*_*l*_← extract sub-volumes from *D*_*u*_ according to *S*

#### Semi-supervised neuron segmentation via mixed-view consistency regularization

In this subsection, we introduce IIC-Net, a semi-supervised framework built upon axial-through spatial mixing and mixed-view consistency regularization (Figure 1C).

##### Axial-through spatial mixing

The affinity graph used to characterize voxel connectivity is generated from dense instance annotations. Their values range between 0 and 1, which indicates whether two associated voxels belong to the same instance (1) or not (0) along the *z*/*x*/*y*- axis. Let two patches with their affinity graphs be {(*l*_*i*_,*y*_*i*_),(*l*_*j*_,*y*_*j*_)}, where $l_{i},l_{j}\in R^{D\times H\times W}$ and *y*_*i*_,*y*_*j*_∈{0,1}^*C*×*D*×*H*×*W*^. Spatial mixing for raw patches and the affinity graph are denoted as M^*R*^(·) and M^*A*^(·), respectively. Large-scale volume EM data often exhibits strong anisotropy,^5^^,^^6^ where the *z*-axis resolution is much lower than that of the *x*- and *y*-axes. To avoid semantic ambiguity caused by asymmetric axial and tangential resolution, we employ an axial-through masking strategy in spatial mixing (Figure 2). The mixed result for *l*_*i*_ and *l*_*j*_ is given by

(Equation 4) $$M^{R}\left(l_{i},l_{j}\right)=\Pi^{R}⊙l_{i}+\left(1-\Pi^{R}\right)⊙l_{j}\text{,}$$

Here, ⊙ denotes Hadamard product, and Π^*R*^∈{0,1}^*D*×*H*×*W*^ is a binary matrix determined by the mixing strategy. Each element of Π^*R*^ indicates whether the voxel comes from *l*_*i*_ (1) or *l*_*j*_ (0). The size of the zero-value region(s) in Π is *D*×*βH*×*βw*, where *β*∈(0,1). The mixed result for the affinity graph *y*_*i*_ and *y*_*j*_ is computed by

(Equation 5) $$M^{A}\left(y_{i},y_{j}\right)=\Pi^{A}⊙y_{i}+\left(1-\Pi^{A}\right)⊙y_{j}\text{,}$$

where $\Pi_{c,d,h,w}^{A}\equiv \Pi_{d,h,w}^{R}$, indicating that the same mixing operation is performed on each channel of the affinity graph.

##### Mixed-view consistency regularization

As network design is not the primary focus of this work, we adopt a modified 3D U-Net as the segmentation backbone *S*(·). This architecture has been widely applied in neuron segmentation.^9^^,^^11^^,^^14^ During training, we randomly sample two labeled pairs {(*l*_1_,*y*_1_),(*l*_2_,*y*_2_)} from *D*_*l*_ and two unlabeled volumes {*u*_1_,*u*_2_} from *D*_*u*_. Let M_*i*_ denote the mixing operation associated with a binary mask Π_*i*_. Prior to mixing, we apply several random data augmentations to increase the diversity of input views, such as Gaussian blurring, additive Gaussian noise, random masking, and photometric perturbations.

Specifically, to maximize the use of limited annotations and support network adaptation, we employ an **intra-dataset mixing** strategy within both labeled and unlabeled datasets (Figure 1C). For labeled data, the supervised loss $L_{Intra}^{L}$ is defined as

(Equation 6) $$L_{Intra}^{L}=L_{CE}\left(S\left(M_{1}^{R}\left({\tilde{l}}_{1},{\tilde{l}}_{2}\right)\right),M_{1}^{A}\left(y_{1},y_{2}\right)\right)\text{,}$$

where $L_{CE}$ represents cross entropy loss, ${\tilde{l}}_{1}$ and ${\tilde{l}}_{2}$ are randomly augmented views of *l*_1_ and *l*_2_, respectively. We leverage unlabeled data by enforcing consistency between predictions of perturbed and original inputs. Specifically, the soft pseudo labels for randomly augmented inputs ${\tilde{u}}_{1}$ and ${\tilde{u}}_{2}$ are computed by *S*(*u*_1_) and *S*(*u*_2_), and thus the mixed-view consistency loss for unlabeled data is given by

(Equation 7) $$L_{Intra}^{U}=L_{SE}\left(S\left(M_{2}^{R}\left({\tilde{u}}_{1},{\tilde{u}}_{2}\right)\right),M_{2}^{A}\left(\underline{S\left(u_{1}\right)},\underline{S\left(u_{2}\right)}\right)\right)\text{,}$$

where $L_{SE}$ represents square error loss.

To enhance feature alignment across datasets, we further perform **inter-dataset mixing**. This operation generates transitional inputs that allow bidirectional information flow and promote shared semantic learning. In detail, the annotated area in the mixed image $M_{3}^{R}\left({\tilde{l}}_{1},{\tilde{u}}_{1}\right)$ is supervised under label map *y*_1_, while the unlabeled area is with the pseudo label *S*(*u*_1_). A binary mask $\Pi_{3}^{A}\in {\left\{0,1\right\}}^{C\times D\times H\times W}$ identifies the source of each voxel, i.e., labeled part (1) or unlabeled part (0). Therefore, the supervision signal of the mixed image can be divided into two parts, denoted as $L_{Inter}^{L}$ and $L_{Inter}^{U}$. Formally,

(Equation 8) $$L_{Inter}^{L}=\Pi_{3}^{A}⊙L_{CE}\left(S\left(M_{3}^{R}\left({\tilde{l}}_{1},{\tilde{u}}_{1}\right)\right),y_{1}\right)\text{,}$$

(Equation 9) $$L_{Inter}^{U}=\left(1-\Pi_{3}^{A}\right)⊙L_{SE}\left(S\left(M_{3}^{R}\left({\tilde{l}}_{1},{\tilde{u}}_{1}\right)\right),\underline{S\left(u_{1}\right)}\right)\text{.}$$

Since expert-annotated masks are generally more accurate than pseudo labels assigned to unlabeled patches, we introduce a weighting coefficient *λ* to balance their contributions in training. The complete loss function is defined as

(Equation 10) $$L_{Semi}=L_{Intra}^{L}+L_{Inter}^{L}+\lambda \left(L_{Intra}^{U}+L_{Inter}^{U}\right)\text{.}$$

Moreover, consistency regularization is effective only when applied to confident predictions on unlabeled data. However, this condition is difficult to achieve when the segmentation network is randomly initialized. To prevent convergence to sub-optimal solutions, we first train the network with $L_{Intra}^{L}$, and then fine-tune it with the complete loss function.

#### Datasets for evaluation

We conduct experimental evaluations on five representative public datasets with diverse neuronal structures, imaging modalities, and spatial resolutions. Due to the variation in anatomical scale and voxel size across datasets, we do not enforce a fixed sub-volume size. Instead, we adopt dataset-specific configurations to balance spatial context and computational efficiency.

##### AC3/AC4

This dataset contains two densely labeled subsets from the Kasthuri dataset.^45^ The original dataset was acquired from the mouse somatosensory cortex using serial section scanning EM at a resolution of 29 nm × 6 nm × 6 nm. AC3 and AC4 consist of 256 and 100 sequential images of size 1024 × 1024, respectively. To ensure reliable evaluation, we divide the datasets into two parts. The bottom 100 layers of AC3 and the complete 100 layers of AC4 are used to simulate unlabeled data *D*_*u*_; the top 100 layers of AC3 are used for testing. The size of the selected sub-volumes (*D*_*l*_) is set as 18 × 380 × 380. Notably, the myelin regions in AC3/AC4 annotations are ignored, leading to confusion in boundary learning. Therefore, we adopt a revised version of annotation for AC4 with myelin annotation (i.e., the training set of SNEMI3D (https://snemi3d.grand-challenge.org/)) and restrict all algorithms to select sub-volumes from AC4 only.

##### CREMI

The CREMI dataset comes from an adult Drosophila brain,^6^ imaged using serial section transmission EM at a resolution of 40 nm × 4 nm × 4 nm. The CREMI dataset comprises three labeled subsets (CREMI-A/B/C), each containing 125 sequential images associated with different regions. Since CREMI-A features a homogenous neuron pattern, our experiments employ the CREMI-B/C dataset. We further divide them into two parts: the top 60 layers of CREMI-B and CREMI-C are considered unlabeled data *D*_*u*_; the bottom 60 layers are used for testing. We select and label sub-volumes of size 18 × 340 × 340. Moreover, the unlabeled set of CREMI, i.e., CREMI-B+/C+, is used for the investigation of distribution mismatch.

##### AxonEM-H

This dataset is imaged using serial section scanning EM from layer 2 in the temporal lobe of an adult human,^5^ containing nine 50 × 512 × 512 sub-volumes at 30 nm × 8 nm × 8 nm resolution. The first six sub-volumes are used as unlabeled data (*D*_*u*_), from which labeled sub-volumes (*D*_*l*_) of size 18 × 340 × 340 are selected, while the rest are reserved for evaluation.

##### Hemi-brain

This dataset is a FIB-SEM volume of the Drosophila melanogaster central brain,^8^ acquired at an isotropic resolution of 8 nm. For subsequent experiments, we utilize six densely labeled volumes provided in the training set, each with a size of 520 × 520 × 520. We partition these volumes into two groups based on their location in the brain. One is designated as unlabeled data (*D*_*u*_), and the other serves as the test data. We select labeled sub-volumes (*D*_*l*_) sized 18 × 300 × 300 from *D*_*u*_.

##### Kasthuri

The size of the Kasthuri dataset is 1850×10747×12895, with a voxel resolution of 29 nm × 6 nm × 6 nm. Our method is evaluated on a sparsely annotated subset cropped from the Kasthuri15 dataset, measuring 300 × 4096 × 4096. The bounding box of this volume ranges from (1050, 6500, 3200) to (1350, 10596, 7296). In the experiments, the top 200 layers serve as unlabeled data (*D*_*u*_), while the bottom 100 layers are designated as the test data. Since the myelin regions are also ignored in official annotations, we select labeled sub-volumes (*D*_*l*_) sized 18 × 380 × 380 from the training set of SNEMI3D.

#### Implementation details

All models are trained on 2 NVIDIA RTX 3090 GPUs using the Adam optimizer. The input patch size is fixed at 18×160×160, the batch size is 4, and the learning rate is set as 1e-4 during unsupervised and semi-supervised training. For selective labeling, the unlabeled volume is partitioned using a sliding window approach with a stride of 8 × 40 × 40. The dimension of the embedding space *L* is 80, and the number of neighbors *K* in CCR is set as 30. For semi-supervised learning, the segmentation network is the residual symmetric U-Net,^9^ which is widely adopted in neuron segmentation. We employ the rectangle and quarter masking strategies in axial-through spatial mixing. The weighting coefficient *λ* for the unlabeled loss component is set to 0.2. The affinity graph has three channels (i.e., *C* = 3), each representing the likelihood between a pixel and its nearest neighbors along the *z*, *x*, and *y* directions.

### Quantification and statistical analysis

#### Evaluation metrics

Two widely-used metrics for neuron segmentation,^11^^,^^12^^,^^13^^,^^14^ the variation of information *VI*^56^ and adapted Rand error ARAND,^57^ are adopted to evaluate the results. *VI* is defined as a sum of the conditional entropies H(·|·) between the segmentation proposal A and the ground truth *T*. It can be further broken down into two components as

(Equation 11) $$\text{VI}={\text{VI}}_{s}+{\text{VI}}_{m}=H\left(A|T\right)+H\left(T|A\right)$$

where VI_*s*_ and VI_*m*_ correspond to split and merge errors, respectively. Let *p*_*ij*_ denote the probability that a randomly selected voxel belongs to segment *i* in *A* and the segment *j* in *T*. Then the ARAND is formally given by

(Equation 12) $$\text{ARAND}=1-\frac{2\sum_{ij}p_{ij}^{2}}{\sum_{i}{\left(\sum_{j}p_{ij}^{2}\right)}^{2}+\sum_{j}{\left(\sum_{i}p_{ij}^{2}\right)}^{2}}$$

Lower values indicate higher segmentation quality in both metrics.
